# Comfortable Meals in an Institution—Tea

**Published:** 1912-07-27

**Authors:** 


### INSTITUTIONAL HOUSEKEEPING.

### Comfortable Meals in an Institution—Tea.

Some slight refection is expected by most people between the two serious events of the day—dinner and supper. It is such a usual custom now in all classes to dine late, that many who are forced by circumstances into institutional surroundings feel the early dinner a serious inconvenience, and are sometimes so unaccommodating they will not adapt themselves to new regulations. We are supposing that all have agreed to keep the appointed rules and make the best of them. Every sensible inmate has dined adequately between the hours of one and two, and suffers no exhaustion from famine when five o'clock draws near. A refreshing cup of tea, with some slight accompaniment, is all that most people desire or expect. Yet even this small incident in the afternoon may be made either a pleasure or a penance. Perhaps tea-time is a moveable feast, and all do not sit down to take it in a regular way, but come in as they are ready, and go out when they like. If this is customary, the tea for late comers will be undrinkable if it has been made in a large urn half-an-hour before. The urn must be replenished, or another with freshly-made tea must be brought in towards the middle of the time, that all may fare comfortably alike. It is better to abandon the use of a large urn altogether and have tea-pots for parties of five, if there is a plentiful supply of boiling water close at hand, that the tea may be made readily as it is wanted. In some institutions each person has a little tea-pot of her own, and an allowance of tea is given out once a week.

The beverage loses all its value unless it can be taken hot and freshly made. The slight refection needed with the refreshing cup should not invariably be bread and butter without variety. There are many other suitable dainties quite as inexpensive and much more acceptable to many. Here is a good opportunity to make use of the dripping, always plentiful in a large establishment. When clarified it can be used to make excellent plain seed-cake or buns, wholesome and delicious, and a welcome variety. Plain currant cake, made with dough and clarified dripping, is far cheaper than buttered bread, and much nicer to the taste of many people. Small pleasures when we are working hard help to diminish the strain of living, and it is far easier to chat merrily over a bit of cake than over the monotonous routine of spreading butter on bread. It is great extravagance, of course, to send in thinly-cut bread and butter for a large party. The plain cake two or three times a week will be appreciated, and the other days can be relieved by the addition of a little jam, or lettuce when in season, or watercress. Such small luxuries are no departure from strict economy, they will not be found to make any difference in the bills in the long run, but they will add to the credit of an institution considerably, and make the inmates feel they are cared for, creating a grateful and home-like atmosphere, where otherwise all would be dreary and comfortless. The appearance on the tea-table of dainty accessories will often put it into the minds of the party to obtain gifts from home of strawberries or preserves for the matron to produce at tea-time, and this is a far better plan than the custom of secret supplies in bedrooms, little cliques of friends making up in private for the deficiencies of the commissariat. Yet sociable tea-parties on birthdays, or other occasions, are not to be discouraged, and the matron who knows how to avoid all favouritism may profitably make use of some little off-room for such little festive gatherings now and then. Those only who live in institutional life can appreciate such breaks in the monotonous routine. Even ten minutes spent informally around a birthday cake can dissipate heavy clouds of depression, and send a worker back to her task merry and buoyant. We are not made to spend every day and every hour exactly like the last; we are creatures of imagination and development, and our simplest actions may be crowned with happy fancy in spite of the most depressing outward conditions.

### Housekeeping Queries.

### Dirty Ceilings.

School Matron.—The ceilings over radiators often become blackened, but not from soot, because there is no smoke rising. The cause of the dirt is that the air over a hot surface becomes heated, consequently lighter, and therefore rises; sometimes even a slight flickering may be noticed. The air is usually dusty, even dirty, and the ceilings are porous, especially in spaces between laths. The dusty air passes through the porous ceiling, which acts as a filtering medium, keeping back the dirt, which gradually accumulates and forms an ever-increasing deposit on the ceilings. Sometimes the filtration of air through ceilings is so marked that the position of the laths above the plaster can be easily traced. We have never yet seen ceiling protectors for use above radiators, but any good firm would be able to supply them to order.

### To Cleanse Blackened Metal.

New Broom.—The housework has evidently been neglected under the old régime. For the blackened brasses try this method of brightening. Dissolve ½ oz. of oxalic acid in a quarter of a pint of boiling water. Add two teaspoonfuls of hydrochloric acid (spirit of salt), shake together, and rub on with a piece of flannel; polish with a dry soft cloth. Note this mixture is a strong poison, so keep it in a red or blue bottle clearly labelled and under lock and key, or make this small amount and throw away any left over after use.

### Saturday Dishes.

Hot-Pot.—4 lb. middle neck or breast of mutton cut into small joints, 5 lb. of potatoes, 2 lb. onions, seasoning, water or stock. Put the meat in an earthenware hot-pot dish or pie-dish or tin in layers with sliced onions and potatoes. Season each layer. Put halved potatoes all over the top. Add one pint of stock. Cover with greased paper. Bake slowly three hours. Take off the paper for the last half-hour. Serve in the dish in which it was cooked. Cost, 2s. 6d.

Vegetable Pie.—One quart of any mixture of cooked vegetables cut in dice, half a pint of thick brown gravy, 1½ oz. of dripping, plain short crust of ¾ lb. of flour. Melt the dripping in a frying-pan, add the vegetables, fry them until slightly browned, add the gravy, season well, and turn into a pie-dish. Prepare the pastry, cover the pie in the usual manner, and bake about half an hour or until the pastry is cooked. The greater the variety of vegetables, the nicer the pie. Macaroni is often mixed in with them, and cooked haricots are a valuable addition. Cost, 1s. 4d.

